# Perseverant, non-indicated treatment of obese patients for obstructive lung disease

**DOI:** 10.1186/1471-2466-13-68

**Published:** 2013-11-22

**Authors:** Spyridon Fortis, Joseph Kittah, Manuel De Aguirre, Maria Plataki, Armand Wolff, Yaw Amoateng-Adjepong, Constantine A Manthous

**Affiliations:** 1 Department of Medicine, Bridgeport Hospital and Yale School of Medicine, New Britain, CT 06050, USA

**Keywords:** Spirometry, Pulmonary function, Obesity, Asthma, Airflow obstruction, Restriction

## Abstract

**Background:**

Bronchodilators are a mainstay of treatment for patients with airflow obstruction. We hypothesized that patients with obesity and no objective documentation of airflow obstruction are inappropriately treated with bronchodilators.

**Methods:**

Spirometric results and medical records of all patients with body mass index >30 kg/m^2^ who were referred for testing between March 2010 and August 2011 were analyzed.

**Results:**

155 patients with mean age of 52.6 ± (SE)1.1 y and BMI of 38.7 ± 0.7 kg/m^2^ were studied. Spirometry showed normal respiratory mechanics in 62 (40%), irreversible airflow obstruction in 36 (23.2%), flows suggestive of restriction in 35 (22.6%), reversible obstruction, suggestive of asthma in 11 (7.1%), and mixed pattern (obstructive and restrictive) in 6 (3.9%). Prior to testing, 45.2% (28 of 62) of patients with normal spirometry were being treated with medications for obstructive lung diseases and 33.9% (21 of 62) continued them despite absence of airflow obstruction on spirometry. 60% (21 of 35) of patients with a restrictive pattern in their spirometry received treatment for obstruction prior to spirometry and 51.4% (18 of 35) continued bronchodilator therapy after spirometric testing. There was no independent association of non-indicated treatment with spirometric results, age, BMI, co-morbidities or smoking history. All patients with airflow obstruction on testing who were receiving bronchodilators before spirometry continued to receive them after testing.

**Conclusion:**

A substantial proportion of patients with obesity referred for pulmonary function testing did not have obstructive lung disease, but were treated nonetheless, before and after spirometry demonstrating absence of airway obstruction.

## Background

Previous studies have demonstrated over-diagnosis of asthma and chronic obstructive pulmonary disease (COPD) in adult populations [1-3]. There are also data to suggest that obese patients are more likely to report respiratory symptoms – especially dyspnea - more than non-obese patients [4]. Accordingly, we hypothesized that obese patients with respiratory complaints prompting pulmonary function tests would be at risk of mischaracterization and persistent, non-indicated treatment of obstructive lung disease.

## Methods

The study protocol was exempted from review as set forth in the Code of Federal Regulations, 45 CFR 46.101(b) by the Bridgeport Hospital Institutional Review Board. Spirometric tests, conducted at Bridgeport Hospital between March 2010 and August 2011, were retrieved from the laboratory’s electronic database. Spirometry was performed according American Thoracic and European Respiratory Society (ATS-ERS) guidelines [5-7] and patients were instructed not to take bronchodilators starting on the night prior to study. Metacholine broncho-provocation tests were performed when requested by the referring physician. Spirometric tests that were not considered acceptable by the reading pulmonologist were not enrolled. Interpretations of pulmonary function tests (PFTs) were performed by American Board of Internal Medicine-certified pulmonologists who applied ATS-ERS standards and guidelines [8]. Per these standards, obstruction is defined as reduced forced expired volume in 1 second: vital capacity (FEV1/VC) ratio below the 5th percentile of the predicted value. Asthma is defined as obstruction *and* increase in FEV1 and/or forced vital capacity (FVC) of ≥12% from baseline in response to bronchodilator, or a positive metacholine challenge test. A restrictive defect is defined as total lung capacity (TLC) below the 5th percentile of the predicted value. In the absence of measured lung volumes, a restrictive ventilatory defect is *suggested* by a reduced VC when FEV1/VC is increased (85–90%) and the flow–volume curve demonstrates a convex pattern. A “mixed pattern” is defined as both FEV1/VC ratio and TLC below the 5th percentiles of their predicted values [8]. In our study, abnormal PFTs that could not be classified in any of the above categories using ATS-ERS standards and guidelines were categorized as inconclusive. Our lab does not measure VC routinely and the FVC was used for interpretation of function tests.

The following data were extracted from electronic medical records for all patients with body mass index (BMI) > 30 kg/m^2^: demographics, co-morbidities, smoking history, pre-testing pulmonary diagnosis, interpretation of spirometry, medications prior to testing, and medications following testing (≥6 months later). Patients were excluded if they had no hospital medical records before and after the spirometry. We followed all the patients for a minimum of 6 months after spirometry. Logistic regression analysis was performed using Epi Info™ to identify variables associated with perseverant treatment of patients with bronchodilators in the absence of airflow obstruction on spirometry. Age, BMI, congestive heart failure (CHF), diabetes mellitus (DM), hypertension (HTN), sex and smoking were chosen as independent variables for modeling based on biological plausibility and/or if they demonstrated an association with inappropriate bronchodilator treatment in univariate analyses.

A P < 0.05 signified statistical significance.

## Results

**Figure 1:**
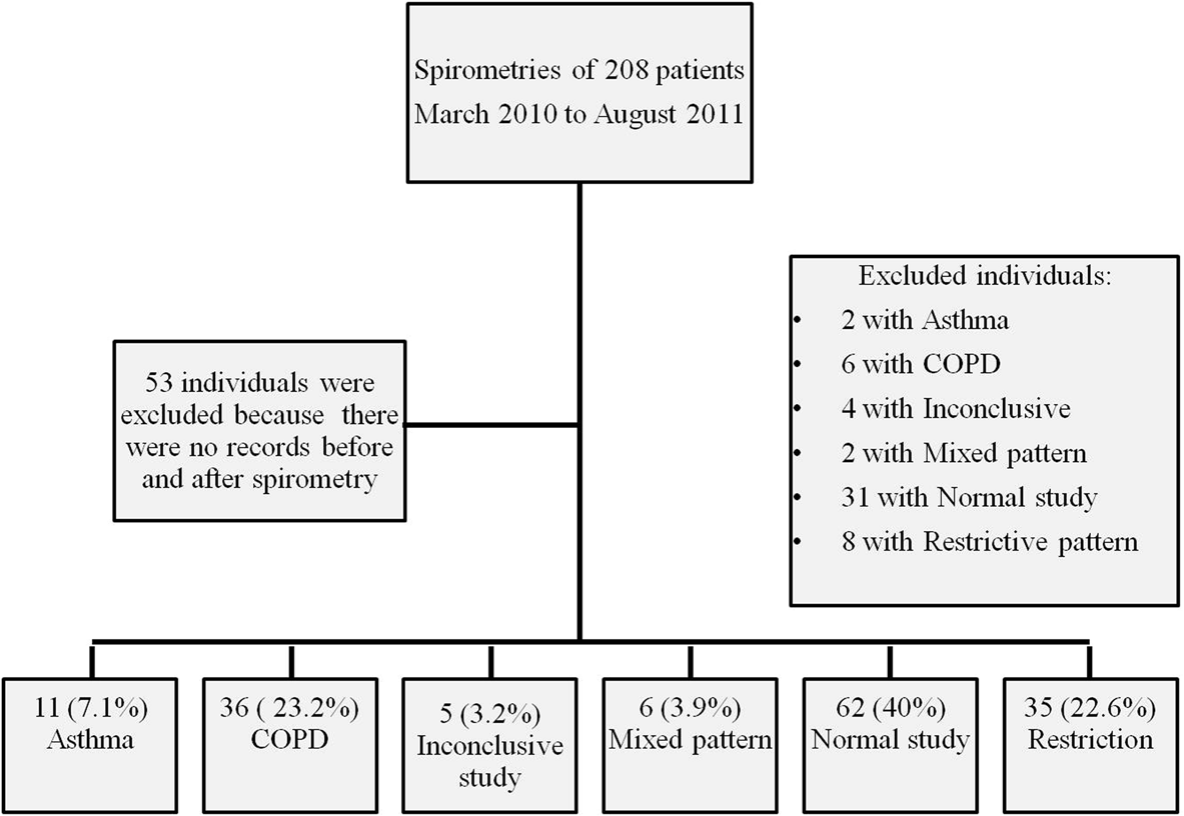
Selection of participants and diagnoses according to pulmonary function tests.

**Figure 2:**
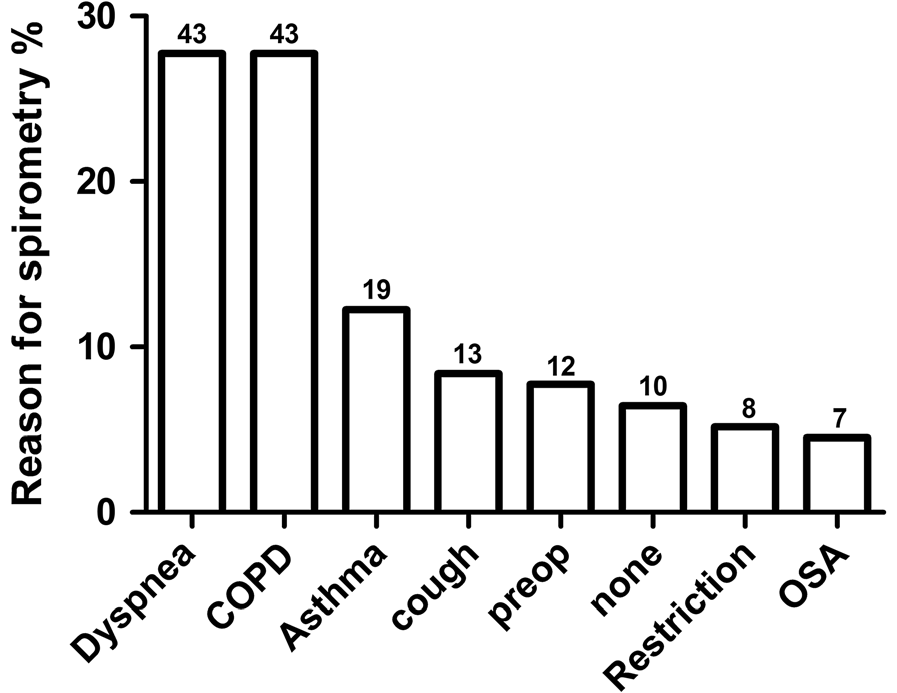
**Reason for the referral to spirometric testing given by referring physicians.** Chronic obstructive lung disease (COPD). Pre-operative evaluation (Preop). OSA (Obstructive sleep apnea).

36 of 244 spirometries were considered technically unacceptable by the reading pulmonologist and were excluded from analysis. 208 patients with BMI > 30 kg/m^2^ had acceptable spirometry during the study period, of whom 53 patients had no electronic records ≥6 months after testing (Figure 1). Of the remaining 155 patients, 84 (55%) were female, mean age was 52.6 ± (SE)1.1 y and BMI was 38.7 ± 0.7 kg/m^2^. 97 (62.8%) had hypertension, 56 (36.1%) diabetes mellitus and 13 (8.4%) congestive heart failure (Table 1). The most common reasons for spirometry were dyspnea (n = 43; 27.7%) and “COPD” (43; 27.7%), followed by “asthma” in (19; 12.3%), cough (13; 8.4%), pre-operative evaluation (12; 7.7%), not specified (10; 6.5%), restrictive lung disease (8; 5.2%) and obstructive sleep apnea (7; 4.5%) (Figure 2). Fifty-seven patients also had measurements of lung volumes (which were interpreted in conjunction with spirometry).

**Table 1:** Characteristics of patients

**Characteristics**	
**Age (years)**	52.6 ± 1.1
**Body mass index (kg/m**^**2**^**)**	38.7 ± 0.7
**Female**	84 (54.8%)
**Hypertension**	97 (62.8%)
**Diabetes mellitus**	56 (36.1%)
**Congestive heart failure**	13 (8.4%)
**Active smokers**	48 (23.1%)

Age and body mass index are presented as means and standard error. Gender, co-morbidities and smoking habit are presented in numbers and percentages of the study population.

Patients demonstrated a mean FEV1 of 66.6 ± 1.4%, mean FVC of 65.9 ± 1.3% with an average FEV1/FVC ratio of 77.8% and a TLC of 82.7 ± 3.7% of the predicted values (Table 2). Please refer to Table 2 for lung function descriptive statistics for each group.

**Table 2:** Lung function descriptive statistics

	**Total**	**Asthma**	**COPD**	**Mixed**	**Normal**	**Restriction**
**FEV1%**	66.6 ± 1.4	59.9 ± 6.4	55.2 ± 2.5	50.1 ± 5.7	92.8 ± 1.6	66.6 ± 2.8
**FVC%**	65.9 ± 1.3	74.3 ± 5.3	72.8 ± 2.6	61.5 ± 7.6	92.5 ± 1.6	65.9 ± 2.7
**FEV1/FVC%**	77.8 ± 1.1	64 ± 5.5	58.9 ± 1.9	64.8 ± 3.5	81.5 ± 0.9	77.8 ± 2.4
TLC%*	82.7 ± 3.7	97.2 ± 6.4	101.5 ± 7.6	66.6 ± 4.6	100.2 ± 13.4	69.1 ± 1.4

Values are presented as means and standard error. FEV1, FVC and TLC are expressed as percentages of the predicted values. *FEV1*, forced expired volume in 1 second; *FVC*, forced vital capacity, *TLC*, total lung capacity. *TLC was available for only 57 patients.

**Figure 3:**
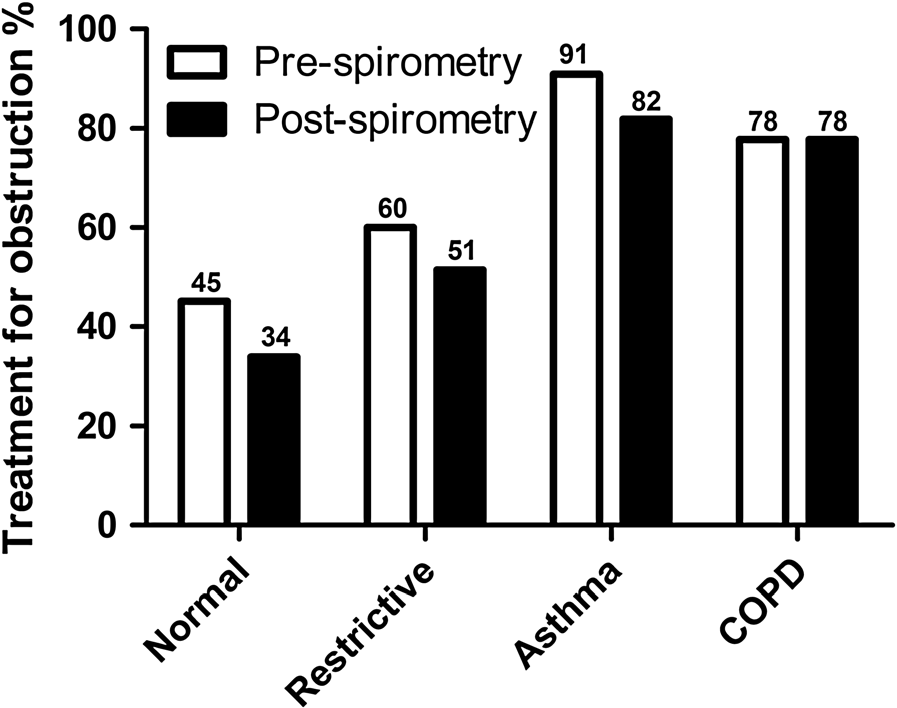
Rate of treatment for obstruction in patients with normal and restrictive, irreversible airflow obstruction (COPD) and reversible obstruction (asthma) before (white bars) and after (black bars) spirometry.

Pulmonary testing was normal in 62 (40%), showed irreversible airflow obstruction in 36 (23.2%), restriction in 35 (22.6%), reversible obstruction suggestive of asthma in 11 (7.1%), mixed pattern (obstructive and restrictive) in 6 (3.9%) and inconclusive studies in 5 (3.2%) patients (Figure 1). Prior to testing, 45.5% (28 of 62) of patients with normal spirometry were being treated with medications for obstructive lung diseases; 15 (53.6%) and 13 (46.4%) of these 28 individuals were misdiagnosed with asthma and COPD, respectively. 33.9% (21 of 62) of these patients continued treatments with medications for obstructive lung diseases despite absence of airflow obstruction on spirometry. 60% (21 of 35) of patients with a restrictive pattern in their spirometry received treatment for obstruction prior to spirometry; 10 (47.6%) and 6 (25.6%) of these patients were misdiagnosed as asthma and COPD, respectively. And 86% continued bronchodilator therapy despite spirometry results that did not demonstrate airway obstruction (Figure 3).

There was no independent association of non-indicated treatment with spirometric results, age, BMI, co-morbidities or smoking (Table 3). All patients with airflow obstruction on testing who were receiving bronchodilators before spirometry (28 of 36 with irreversible and 9 of 11 with reversible obstruction) continued to receive them after testing (Figure 3). Medications used to treat patients with normal tests included beta2-agonists in 40.3% (25 of 62), inhaled corticosteroids in 16.1% (10), anticholinergics in 14.5% (9), systemic corticosteroids in 8.1% (5) and leukotriene modifier in 1.6% (1). Individuals with restrictive pattern were prescribed beta-2 agonists in 54.3% (19 of 35), inhaled steroids in 28.6% (10), anticholinergics in 14.3% (5), and (short-course) systemic corticosteroids 14.3% (5) (Table 4).

**Table 3:** Adjusted odds ratios (ORs) and 95% confidence intervals (CIs) for determinants of treatment for obstructive airway disease among individuals with normal and restrictive pattern in their PFTs

**Spirometry**	**Normal**			**Restriction**		
**Term**	**OR**	**95% C.I.**	**P-Value**	**OR**	**95% C.I.**	**P-Value**
**Age**	1.00	0.95-1.04	0.87	1.10	1.00-1.21	0.05
**BMI**	0.97	0.90-1.05	0.51	1.10	0.98-1.22	0.10
**CHF**	2.24	0.16-30.66	0.54	0.18	0.02-1.87	0.15
**DM**	2.05	0.59-7.15	0.26	0.07	0.00-1.77	0.11
**HTN**	0.54	0.16-1.89	0.34	1.6x10^6^	0.00-1x10^12^	0.96
**Sex**	1.43	0.42-4.89	0.57	2.36	0.24-23.72	0.46
**Smoking**	1.63	0.44-6.00	0.47	1.89	0.24-15.10	0.55

Adjustments were made for clinically relevant discriminators such as age, body mass index (BMI), congestive heart failure (CHF), diabetes mellitus (DM), hypertension (HTN), sex and smoking.

**Table 4:** Treatment individuals received before spirometry

	**Normal (62)**	**Restriction (35)**	**COPD (36)**	**Asthma (11)**
**Inhaled Beta**_**2**_**-agonists**	40.3% (25)	54.3% (19)	69.4% (25)	72.7% (8)
**Inhaled corticosteroids**	16.1% (10)	28.6% (10)	38.9% (14)	63.6% (7)
**Inhaled Anticholinergic**	14.5% (9)	14.3% (5)	33.3% (12)	9.1% (1)
**Oral corticosteroids**	8.1% (5)	14.3% (5)	13.9% (5)	9.1% (1)
**Leukotriene antagonists**	1.6% (1)	0	5.6% (2)	18.2% (2)

## Discussion

This observational cohort study demonstrates that half of all obese patients referred for spirometry were treated empirically with bronchodilators before testing, and that even after spirometry demonstrated the absence of airflow obstruction 40% (39 of 97) continued to be prescribed therapies directed at obstructive lung disease 6 or more months after testing. Misdiagnosis of obstructive lung disease is not uncommon. Lindesmith and colleagues reported that many Canadian community clinicians diagnosed obstructive lung disease on clinical grounds; 41% of (37 of 90) patients were labeled as asthmatic but failed to meet diagnostic criteria [2]. As in our study, 62% of these misdiagnosed patients continued to receive inhaled beta-agonists, while 43% received inhaled glucocorticoids. In two separate studies, 30% of patients diagnosed with asthma on clinical grounds did not satisfy pulmonary function test criteria for obstruction [3,9]. In another study, 10-41% of patients in primary care offices used inhaled steroids for a clinical diagnosis of asthma or COPD without spirometric evidence to support the diagnoses [1].

We are unaware of a previous study that has focused on obese patients receiving spirometric testing. We focused on this population because they comprise a large demographic who commonly present with respiratory complaints [4], most often related to restrictive respiratory mechanics [10] and increased oxygen cost of breathing [4], and who might be – at least in theory – more vulnerable to complications of unnecessary polypharmacy. While some studies have suggested an inverse relationship of BMI and FEV1 [11,12] and an association of obesity and obstructive lung diseases [13,14], the frequency with which dyspnea is caused by obstructive vs. restrictive physiology in obese patients has not been well-studied. Obese patients are more likely to report respiratory symptoms – especially dyspnea -more than non-obese patients [4]. Truncal obesity can reduce chest wall compliance, and respiratory muscle strength and function [15]. Accordingly, we hypothesized that obese patients with respiratory complaints prompting pulmonary function tests would be at risk of mischaracterization and persistent, non-indicated treatment of obstructive lung disease. Our results supported the hypothesis, but most surprising, treatments with medications for obstruction were continued without clinical or spirometric indications. Clearly such patients are exposed to complications and costs of these therapies without proven or plausible clinical benefits. Interestingly, the rate of bronchodilator use before and after PFTs remained the same in patients with COPD but decreased in those with asthma. We suspect this is a statistical artifact related to small sample size, nonetheless, it is a perseverant treatment. However, it could suggest bronchodilator prescription driven inappropriately by symptoms (i.e. dyspnea) rather than objective physiologic abnormalities.

We did not examine why clinicians continue to prescribe bronchodilators to patients whose function tests did not show obstruction. However, this phenomenon has been noted with other medications, most notably acid-suppressors. For example, Slain and colleagues demonstrated that 32 of 121 adult patients admitted to hospital reported taking either proton pump inhibitors or histamine-blocking agents [16]. Similarly, 62 of 213 patients admitted to the University of Michigan Hospital wards were receiving acid suppressors prior to admission, which increased to 152 of 213 during admission (only 15 “indicated”), and 115 of 213 were discharged on these medications [17]. The concept of “therapeutic inertia” has been introduced to describe situations in which clinicians fail to treat despite evidence of disease [18]. It is possible that perseveration of non-indicated acid suppression following hospitalization and bronchodilators without spirometric evidence of obstruction represent therapeutic inertia. Alternatively, perhaps this is a result of treatment bias; for example, if patients experience a placebo effect, there may be an inclination to continue a medication even without objective evidence of improvement.

Medication errors are a common cause of harm in hospitalized patients [19], and so appropriately the focus of regulatory scrutiny [20]. While perseverant treatment of our non-obstructed obese patients might be regarded as a “medication error,” the genesis is probably more a malady of “systems-based practice.” Too often disease entities are entered in the medical record without meeting firm diagnostic criteria; sometimes on the basis of medications administered presumptively [1-3]. Tests may be ordered but not checked or carefully considered to ensure that treatment regimens are appropriate [21]. Researchers are beginning to develop taxonomies for these diagnostic and therapeutic errors [21]. Arguably, the most important message is that diagnoses should not be taken “at face value” and perpetuated in medical records. Perhaps, reconciliation of medications *and* diagnoses will promote safer, patient-centered care. Specifically, our data combine with other studies [9,22] to suggest that obesity is not – in itself – an obstructive, but rather is more commonly a restrictive lung disease. While obstructions may occur dynamically with sleep in these patients, and some obese patients may have true asthma or smoking-related obstructive disease, clinicians should exercise greater caution – via confirmatory spirometry – before assigning or perpetuating the label of obstructive lung disease to obese patients.

Mislabeling or misdiagnosis is not without risks and costs, especially when (these) patients receive medications that can cause complications but provide no proven benefit (since they did not have obstruction). In addition to tremor, tachycardia and hypokalemia, beta-agonists have been associated with increased mortality in asthmatic patients, especially African Americans [23]. Anticholinergic medications may also increase the risk of cardiovascular death [24]. Inhaled and systemic corticosteroids are associated with diabetes, hypertension, infection, pneumonia, glaucoma, adrenal insufficiency, thrush, dysphonia, myopathy, and cardiovascular events [25]. While we can find no suggestion that obese patients are more vulnerable to complications from these therapies, they could exacerbate some diseases (e.g. hypertension, diabetes) that are more common in the obese population.

Potential harm aside, our study has substantial financial implications. 33% [26] of 246 million American adults [27] are overweight or obese. If half of the 2% of those with BMI > 22.1 kg/m^2^ who report asthma and take bronchodilators [22] really don’t have reversible airflow obstruction, substantial unnecessary cost (and risks) accrues. At an average of $100/year (for generic albuterol) [28], the total unnecessary cost – just for medication – is over $80 million in the U.S. If more expensive medications are administered – tiotropium and salmeterol/fluticasone cost over $1000/year [29]– unnecessary cost increases accordingly.

Our study has several limitations including its small sample size and sampling bias since our cohort represents only the subgroup of obese patients referred for pulmonary function testing. Since this study was conducted at only one hospital, these results should be generalized cautiously. But there is abundant evidence to suggest that incorrect diagnosis (and subsequent treatment) of obstructive lung disease occurs more globally [1-3,9]. Obese patients are not unique; but rather our results emphasize that pulmonary testing should be used to confirm or refute clinical impressions, and to guide appropriate management. In addition, lung volumes were not measured in 63% of patients. Vital capacity may differ from FVC in patients with substantial airway obstruction [30]. While airway obstruction was rare in our study population, this methodologic limitation, inherent in our retrospective study design, could reduce the precision of our conclusions. Another limitation of our study is that most (all but 2) individuals with normal spirometry did not have metacholine challenge to rule out bronchial hyper-responsiveness. In addition, although patients were instructed not to use bronchodilators for >12 hours, we did not ascertain the rate of compliance. However, that does not undermine the importance of our findings, since patients continued to receive bronchodilators even after function tests failed to demonstrate airflow obstruction. We cannot assert with certainty that bronchodilators were administered continuously/daily in all patients without indications in the follow-up period (i.e. in some the medications could have been stopped and later restarted for a bronchospastic episode that was not documented in our medical records). It is also possible that aerosols were administered to our patients for indications other than obstructive symptoms e.g. to improve mucociliary clearance [31]. However, since aerosols are seldom used solely for this indication in clinical practice, it is not unreasonable to assume that clinicians continued aerosols for (misdiagnosed) obstructive lung disease.

## Conclusion

In conclusion, 45.2% (28 of 62) of obese individuals with normal PFTs and 60% (21 of 35) with restrictive pattern received inappropriate treatment for obstructive lung disease prior to PFTs. Fully a third (33.9%; 21 of 62) of these obese patients with normal lung mechanics, and half (51.4%; 18 of 35) with purely restrictive defects, continued to receive inappropriate treatment after function testing. A substantial number of individuals also received anticholinergics and inhaled and systemic steroids. Inappropriate use of these medications exposes patients to risks of complications and increases the cost of care with no proven or theoretical benefit.

## Competing interests

The authors declare that they have no competing interests.

## Authors’ contributions

SF participated in study design, data acquisition, data analysis, and manuscript preparation; CM and AW in study design and preparation of the manuscript; DA, JK and PM in data acquisition; AA in study design and data analysis. All authors approved the final content of the article.

## Pre-publication history

The pre-publication history for this paper can be accessed here:

http://www.biomedcentral.com/1471-2466/13/68/prepub
